# Predicting COVID-19 positivity and hospitalization with multi-scale graph neural networks

**DOI:** 10.1038/s41598-023-31222-6

**Published:** 2023-03-31

**Authors:** Konstantinos Skianis, Giannis Nikolentzos, Benoit Gallix, Rodolphe Thiebaut, Georgios Exarchakis

**Affiliations:** 1 BLUAI, Athens, Greece; 2 École Polytechnique, Palaiseau, France; 3 IHU, Strasbourg, France; 4 ICube, CNRS, University of Strasbourg, Strasbourg, France; 5 INSERM U1219, Inria SISTM, University of Bordeaux, Bordeaux, France; 6 Pôle de Santé Publique, Service d’Information Médicale, CHU de Bordeaux, Bordeaux, France

**Keywords:** Computer science, Epidemiology, Health policy

## Abstract

The pandemic of COVID-19 is undoubtedly one of the biggest challenges for modern healthcare. In order to analyze the spatio-temporal aspects of the spread of COVID-19, technology has helped us to track, identify and store information regarding positivity and hospitalization, across different levels of municipal entities. In this work, we present a method for predicting the number of positive and hospitalized cases via a novel multi-scale graph neural network, integrating information from fine-scale geographical zones of a few thousand inhabitants. By leveraging population mobility data and other features, the model utilizes message passing to model interaction between areas. Our proposed model manages to outperform baselines and deep learning models, presenting low errors in both prediction tasks. We specifically point out the importance of our contribution in predicting hospitalization since hospitals became critical infrastructure during the pandemic. To the best of our knowledge, this is the first work to exploit high-resolution spatio-temporal data in a multi-scale manner, incorporating additional knowledge, such as vaccination rates and population mobility data. We believe that our method may improve future estimations of positivity and hospitalization, which is crucial for healthcare planning.

## Introduction

Accurately modeling contagion dynamics is of paramount importance for preventing or controlling outbreaks of infectious diseases. Thus, it comes as no surprise that a variety of models incorporating sophisticated contagion mechanisms have been proposed over the past years ^1–3^. These models have contributed to the design of better public health policies since they allow us to improve our understanding of how infectious diseases spread. However, these models are not always accurate and several challenges still need to be unresolved ^4,5^. This became particularly evident during the worldwide pandemic of COVID-19. The SARS-CoV-2 virus which started spreading in Wuhan, China in late 2019, spread to most countries around the world within a few months causing the pandemic of the COVID-19 disease. The virus turned out to be particularly contagious. As of February 14, 2022, a total of 5,783,776 deaths and 404,910,528 cases of COVID-19 were confirmed worldwide ^6^. The COVID-19 pandemic has had a significant impact not only on the lives of individuals, but also on the global economy ^7^ and on the environment ^8^, among others.

During a pandemic, it is critical for governments, policymakers and public health agencies to accurately predict the spread of the infection. Among others, this will allow them to impose measures that will successfully reduce the spread of the virus and also to effectively allocate healthcare resources. The standard approach to predicting the spread of an infection is to use mathematical models such as the ones mentioned above. Several such models have been developed for COVID-19 ^9–13^. There even exist models for determining the cost and economic health outcomes of government interventions ^14^. However, such models usually integrate a limited number of mechanisms and thus do not fully capture the complex nature of contagion dynamics. These models can be made more complex by adding more detailed and sophisticated mechanisms. However, in many cases, this requires a lot of effort or is practically not feasible. Furthermore, more complex models usually comprise of a large number of parameters whose values can be difficult to infer from limited data.

Recently, there has been an increasing interest in machine learning and artificial intelligence approaches to address the limitations of mathematical models ^15,16^. This approach involves training predictive models on collected real-world data. Such data-driven models can then be used for making accurate predictions, but also for gaining insights into complex phenomena. Although these approaches were originally applied to other areas of physics ^17^, they have recently started being applied to epidemics spreading perhaps also motivated by the outbreak of COVID-19 ^18–20^. Among the different families of deep learning approaches that have been developed in the past years, graph neural networks (GNNs) ^21^ are particularly suited to problems that involve some kind of network structure. These models have been applied with great success to different problems such as web recommendations ^22^ and modeling physical systems ^23^. Thus, GNNs could offer great potential to build effective data-driven dynamical models on networks. Ideally, if we had access to the social network of all individuals which captures the interactions between them, we could build a model of the spread of the disease. Unfortunately, for the case of COVID-19, such information is not available.

In the absence of the aforementioned data, we use population mobility data instead. It has been reported that population movement between regions has a significant impact on the spread of the disease ^24^. Previous studies have also investigated what is the impact of other factors on the spread of COVID-19 such as the use of protective masks ^25^. By presenting a model with the amount of people that moved from one place to another, we can approximate, in a sense, the amount of people from the first region that came into contact with people from the second region. Mobility data can be represented as a graph where nodes correspond to regions and edges model the mobility between regions. Thus, the above becomes a well-suited setting for GNNs. Therefore, in this work, we show that GNNs can be used to model contagion dynamics on complex networks, i. e., networks that model mobility patterns. One of the key concepts behind GNNs is the concept of message passing. Specifically, there is a vector representation associated with each node of the graph, and this representation is updated for a number of iterations based on messages received from the node’s neighbors. Thus, each node (i. e., region) receives messages that contain information about the spread of the virus in its neighboring nodes (i. e., regions) along with the number of people that arrived from these regions. In this study, we propose a multi-scale approach which uses GNNs to extract features from both low- and high-resolution data, thus leveraging information from different geographic levels. We apply the proposed architecture to the problem of modeling the spread of COVID-19 in France. Our findings indicate that in the context of COVID-19, leveraging additional features in a learning model can increase the model’s predictive ability. For instance, we found that disease spread depends on population mobility since this feature improves the predictive performance of our models. Similar conclusions were drawn for other features such as the vaccination rates. Finally, our analysis showed that the use of more granular data also benefits model performance. Note that COVID-19 spreading is a highly uncertain process which depends on many factors including human behavior, virus mutation, and vaccination rates. Thus, one cannot expect a model to always provide reliable predictions. Overall, this study shows that we can leverage mobility data to produce data-driven models which can complement existing methods.

Finally, it should be mentioned that this is not the first work to use GNNs to predict the spread of COVID-19 ^26–32^. However, there are major differences between these studies and our work. For instance, some studies do not use mobility data ^28^. Others use single-scale models ^26,27,29^, while we Furthermore, most of these works do not focus on France, but on other countries such as the United States ^26,28,31^, Germany ^29^ or Japan ^32^. The datasets employed in these studies span time periods before the start of COVID-19 vaccination in the United States ^26,28^ or ignore or entirely ignore vaccination rates ^30–32^. On the other hand, in our work, we also consider the impact of vaccination on the spread of the disease.

## Data

**Figure 1:**
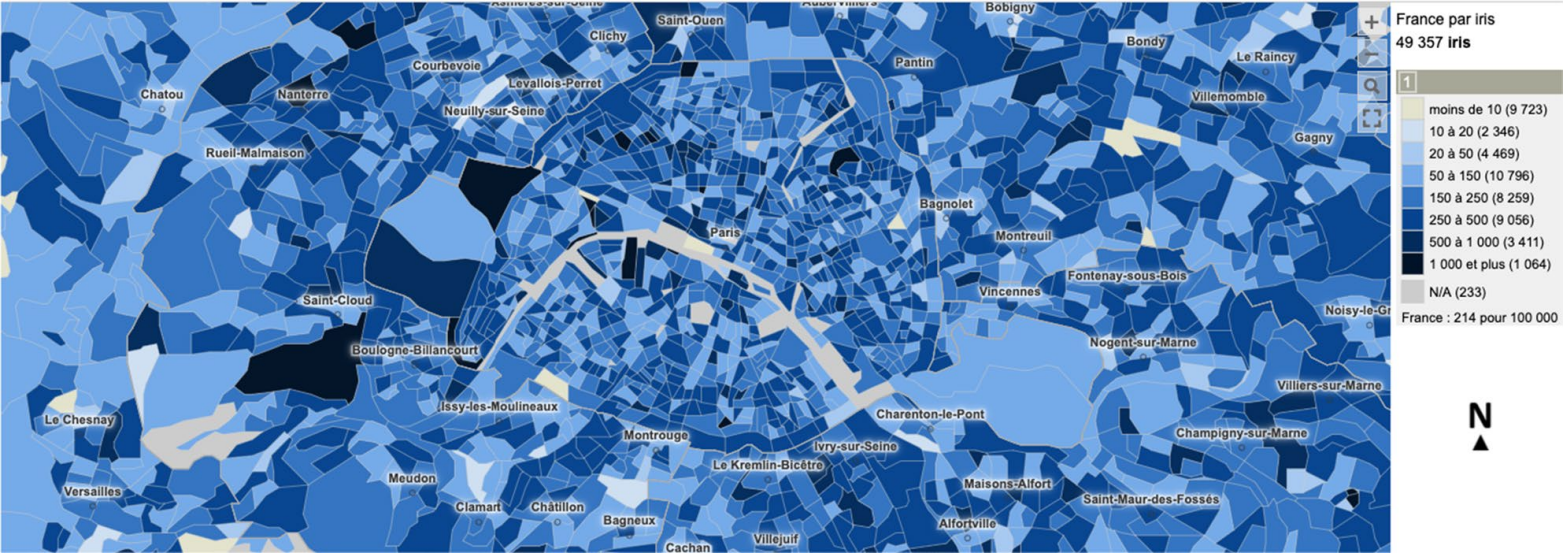
IRIS-level positive cases in Paris (27-01-2021 to 02-02-2021), visualized via the Géodes tool^33^.

**Table 1:** Statistics of the dataset used in this study.

Dataset		
Time Span	27/12/2020–27/6/2021	
Level	Department	Iris
Regions	94	30,378
Avg positive cases	180.65	–
Avg hospitalized patients	12.81	–

### COVID-19 positivity and hospitalization datasets

Modeling the spread and predicting COVID-19 positivity and hospitalization requires high-quality data, which are freely available by Santé publique France. During the health crisis linked to the COVID-19 epidemic, Santé publique France is responsible for monitoring and understanding the dynamics of the epidemic, anticipating the various scenarios and implementing actions to prevent and limit the transmission of this virus on the national territory. Santé publique France is the french national public health agency, created in May 2016. Towards this goal, the organization is gathering and offering freely numerous datasets that provide information regarding the COVID-19 pandemic. The virological surveillance indicators come from the screening information system (SI-DEP), the objective of which is to report data from tests (RT-PCR) carried out by all city laboratories and hospitals concerning the SARS-COV-2.

**Figure 2:**
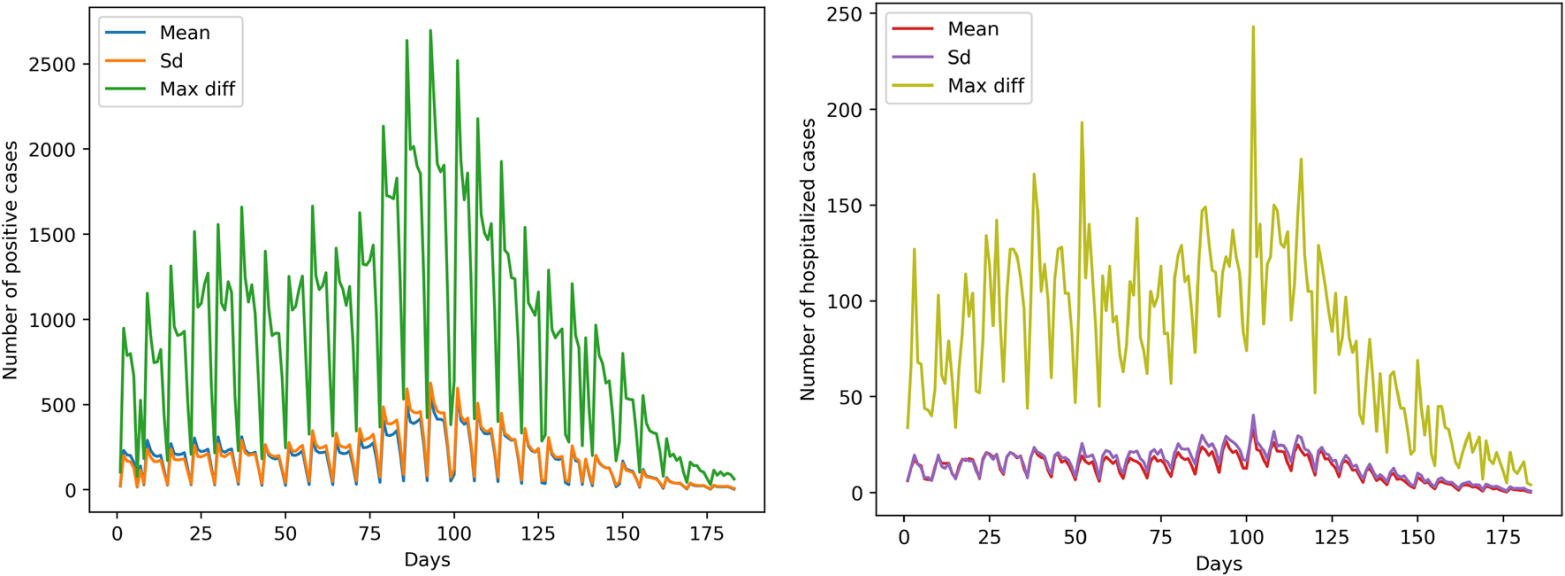
Mean, standard deviation and maximum difference of positive (left) and hospitalized cases (right) per day.

Regarding positivity, data are available in both department and IRIS scale. IRIS represents the fundamental unit for dissemination of infra-municipal data. These units respect geographic and demographic criteria, while having clearly identifiable borders. The dataset provides information either in absolute numbers for departments, or about the class of positivity rate for all ages over 7 rolling days for IRIS areas. Concerning IRIS data, exact rates are not displayed in order to avoid identification of people tested, especially those who test positive. A screenshot of the Géodes tool^33^, created by Santé publique France, with regards to IRIS areas, is shown in Fig. 1. The positivity rate corresponds to the number of positive tests compared to the number of tests carried out. For the task of predicting hospitalization, we used the absolute number of new patients hospitalized in the last 24 h. Information and statistics on the data can be found in Table 1, while a visualization of the spread’s behavior throughout the studied duration is presented in Fig. 2. All data are accessible online ^34^.

### Facebook mobility dataset

In order to study the spread of a virus like COVID-19, we need to track population movement, as it is one the most important factors. Responding to the COVID-19 outbreak, technology companies and mobile phone operators are coming forward to provide important, epidemiologically-relevant data from mobile devices, that could help to inform policies that reduce the spread of the virus. As stated in^35^, research and public health response communities can and should use population mobility data, with appropriate legal, organizational, and computational safeguards in place. After aggregation, these data can help refine interventions by providing near real-time information about changes in patterns of human movement. An example of these mobility datasets is provided by Facebook and this is the dataset we employed in this study ^36^. Besides mobile phone applications, other major sources of mobility data include public transit systems and mobile operators ^37^.

### Vaccination dataset

Another significant factor that could help to study the spread of COVID-19, would be to look at vaccination percentages of each department. Vaccination can be crucial to block transmission and prevent severe hospitalization. As our study is focused in the first 6 months of 2021, we only incorporate information about 1-dose vaccinations. Vaccination information was integrated as additional node features. The dataset is publicly available ^38^.

## Results

As already discussed, in this paper, we study the evolution of COVID-19 in France, and we thus train the proposed model on data collected from this country. Specifically, among the different administrative divisions of France (i. e., administrative regions, departments, communes), we focus on departments since this is the finest (in terms of granularity) administrative division for which both the daily number of new cases and mobility data are available. In fact, departments lie in between administrative regions and communes; in our case we extract data for 93 departments in metropolitan France in total.

**Figure 3:**
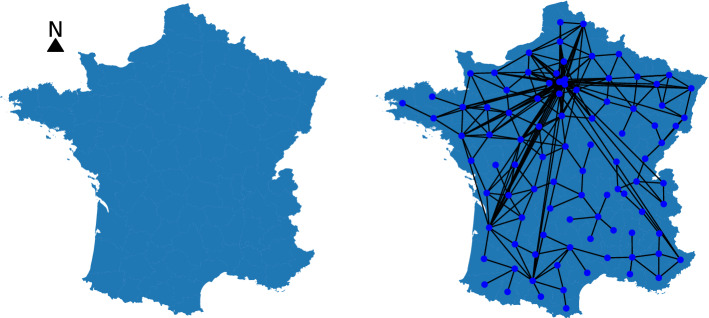
A map of the departments of metropolitan France (left). A graph where nodes represent departments of metropolitan France and edges capture mobility patterns (right). Two nodes (i. e., departments) are connected by an edge if people have moved from the one department to the another. Such graphs constructed from mobility data are given as input to the GNN model.

We represent the whole country as a graph $G=(V,E)$ where $n=|V|$ denotes the number of nodes and $m=|E|$ denotes the number of edges, as shown in Fig. 3. Nodes represent departments, while edges model mobility patterns. The weight of an edge denotes the total number of people that moved from one department to another department. Note that graph *G* can also contain self-loops which correspond to the mobility behavior within the departments. Furthermore, the nodes of graph *G* are annotated with attributes. Therefore, each vertex *v* in the graph is associated with a feature vector $\textbf{h}_v^{(0)} \in \mathbb {R}^d$ where *d* is the feature dimensionality. We use the following features for a node *v* (representing some department): (1) population of department; (2) number of cases for each one of the previous 7 days; (3) vaccination rate; and (4) volume of COVID-19 hospitalizations for each one of the previous 7 days (only for models trained to predict number of hospitalizations). Since the quantity of interest is the daily number of new cases, we do not create just a single graph, but a series of graphs, each corresponding to a specific day *t*, i. e., $G^{(1)}, \ldots , G^{(T)}$.

Before presenting the results about the predictive ability of the proposed approach, we provide some intuition for why GNNs could be of interest to the study of disease spread across a country. For a given day, we assume that the total number of positive cases in a region is a function of the number of cases reported for that region in the previous days, the region’s population, the number of vaccinated individuals residing in the region and the population mobility both within the region and across regions. If the nodes (i. e., regions) of the graph are annotated with the first three features described above, then GNN models that employ message passing schemes can approximate the above function. Indeed, given a node and its neighbors, these models can learn to combine features received by the neighbors which contain information about the spreading status of those regions along with the spreading status of the region itself.

In our experiments, we investigate how accurate the proposed model is in predicting the number of new cases per department as well as the number of hospitalized patients with COVID-19. Our experimental protocol is similar to that of prior work ^27^. Specifically, we train the models using data from day 1 to day *T* , and then use the model to predict the number of cases (or hospitalized patients) for each one of the next $\tau$ days. Thus, we make predictions for all days between $T+1$ and $T+\tau$. Following prior work ^27^, we evaluate the effectiveness of the model in short-, mid- and long-term predictions. Therefore, we consider three different time resolutions by setting $\tau$ to 3, 7 and 14, respectively. We expect the model to make more accurate short-term predictions than long-term predictions. It should be mentioned that we do not train a single model that predicts the number of cases (or hospitalizations) for all days from $T+1$ to $T+\tau$. On the other hand, we train a different model to make predictions for days $T + i$ and $T + j$ where $0 < i, j \le \tau$ and $i \ne j$. Thus, there are $\tau$ different models, and each model focuses on a single day between $T+1$ and $T+\tau$. Note also that we do not consider a single task, but multiple tasks instead. Formally, let *K* be the total number of days for which data is available (in our case, *K* is equal to 182 days (from 27/12/2020 to 27/06/2021)). Let also $\mathcal {T}$ the denote the minimum size of the training set. Then, we define $K-\tau -\mathcal {T}+1$ tasks in total. For the first task, the training set consists of all days between 1 and $\mathcal {T}$ (i. e., $T=\mathcal {T}$). Likewise, for the second task, the training set consists of all days between 1 and $\mathcal {T}+1$ (i. e., $T=\mathcal {T}+1$). For the last task, the training set consists of days from 1 to $T=K-\tau$.

For a given task, to evaluate the performance of a model, we compute the mean absolute error which compares the predicted total number of cases (or hospitalized individuals) in each department against the corresponding ground truth, throughout the test set:

1 $$\begin{aligned} \text {error} = \frac{1}{n \tau }\sum _{t=T+1}^{T+\tau }\sum _{v\in V}|\hat{y}^{(t)}_v - y^{(t)}_v| \end{aligned}$$

where $y^{(t)}_v$ denotes the number of cases (or hospitalized individuals) in the department represented by node *v* on day *t*, and $\hat{y}^{(t)}_v$ denotes the predicted number of cases (or hospitalized individuals) for the same department and day. Then, we compute the average of the errors of all tasks.

**Table 2:** Average error for $\tau = \{1,\ldots 3\}$, $\{1, \ldots 7\}$ and $\{1, \ldots 14\}$ (± standard deviation over days of prediction), in number of positive cases per department.

Model	3 days prediction	7 days prediction	14 days prediction
Baselines			
AVG	117.33 ± 0.56	118.44 ± 1.21	121.27 ± 3.14
AVG WINDOW	88.43 ± 0.68	73.18 ± 3.26	87.02 ± 9.27
ARIMA	118.98 ± 0.77	120.02 ± 2.42	121.65 ± 4.77
PROPHET	68.37 ± 1.47	72.82 ± 3.78	93.06 ± 16.0
LSTM	146.00 ± 0.39	147.32 ± 1.21	153.06 ± 1.85
MPNN	71.57 ± 16.6	69.90 ± 9.73	76.25 ± 13.2
MPNN+LSTM	85.23 ± 28.8	91.05 ± 19.2	84.83 ± 16.1
Proposed			
MPNN (vac)	66.72 ± 15.6	66.04 ± 11.0	121.12 ± 22.1
MPNN+LSTM (vac)	83.22 ± 28.3	91.54 ± 20.1	112.49 ± 21.0
Multi-scale MPNN (IRIS)	**57**.**79** ± **11**.**6**	**57**.**27** ± **8**.**96**	**73**.**05** ± **14**.**4**
Multi-scale MPNN (IRIS, vac)	61.54 ± 13.8	63.85 ± 10.7	83.34 ± 15.2

Bold indicates best mode. (vac) stands for using vaccination data.

**Figure 4:**
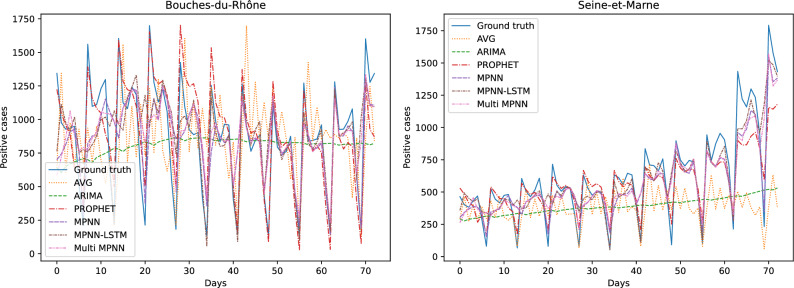
Number of COVID-19 cases per day for two departments of metropolitan France and predicted number of cases by the different approaches.

Table 2 illustrates the average errors in terms of the number of positive cases of the different approaches (along with standard deviations across the different tasks). We use (vac) to denote models that take graphs as input whose nodes are annotated with vaccination rates. We also use Multi-scale to denote models that take as input both department level and IRIS level graphs, i. e., models that combine coarse- and fine-level representations. We can see that our Multi-scale approach is the best performing method on all three considered time resolutions (i. e., $3-$, $7-$ and $14-$days prediction). For short-term and mid-term predictions, instances of the proposed approach achieve almost similar levels of performance as those of PROPHET. For long-term predictions, the difference between the performance of PROPHET and the other methods is quite wide. The two simple baselines that do not involve learning (AVG and AVG WINDOW) outperform common learning models such as ARIMA and LSTM on all three time resolutions. For a specific department and a test day, the AVG baseline predicts a value equal to the average number of cases up to the time of the test day, while the AVG WINDOW baseline predicts a value equal to the average number of cases in the past *d* days (*d* was set equal to 7 in our experiments). They also outperform MPNN+LSTM in some cases. On the other hand, MPNN and its multi-scale variant yield lower average errors than the baselines in all cases. We also observe that adding the vaccination rate as a feature leads to slightly smaller average errors in most cases, while it seems to be more effective for mid-term predictions. The multi-scale variant of the proposed model also leads to performance improvements in all cases. For instance, in the case of the 3 and 7 days ahead forecasts, this model offers MPNN absolute improvements of $13.78\%$ and $12.63\%$ in test error, respectively. Overall, our experiments demonstrate that the proposed approach can serve as a useful state-of-the-art tool for predicting the number of future COVID-19 cases, and thus for combating the spread of the disease. The results that are given in Table 2 measure the average error over the whole prediction horizon and all the 93 considered departments in metropolitan France. Even though the average error summarizes the predictive performance of the different methods, to provide more clear insights about their performance, we chose two out of the 93 departments (Bouches-du-Rhône and Seine-et-Marne) and we visualized the predicted number of cases (one day ahead prediction) for the different approaches. The results are given in Fig. 4. We observe that AVG and ARIMA fail to achieve high levels of performance, while Prophet, MPNN, MPNN-LSTM and Multi-scale MPNN all predict the number of COVID-19 cases with high accuracy. It is hard to determine which model performs the best since the above 4 models provide similar predicted number of cases.

**Table 3:** Average error for $\tau = \{1,\ldots 3\}$, $\{1, \ldots 7\}$ and $\{1, \ldots 14\}$ (± standard deviation over days of prediction), in number of hospitalized patients per department.

Model	3 days prediction	7 days prediction	14 days prediction
Baselines			
AVG	8.23 ± 0.03	8.33 ± 0.10	8.50 ± 0.19
AVG WINDOW	6.13 ± 0.15	5.38 ± 0.16	6.07 ± 0.49
ARIMA	8.51 ± 0.18	8.92 ± 0.29	9.74 ± 0.66
PROPHET	6.09 ± 0.06	6.34 ± 0.17	7.00 ± 0.57
LSTM	7.41 ± 0.01	7.82 ± 0.04	6.98 ± 0.12
MPNN	6.66 ± 0.26	6.10 ± 0.55	5.71 ± 0.46
MPNN+LSTM	5.63 ± 0.58	6.20 ± 0.59	5.77 ± 0.31
Proposed			
MPNN (vac)	6.39 ± 1.13	5.54 ± 0.33	7.23 ± 0.76
MPNN+LSTM (vac)	5.92 ± 0.82	5.65 ± 0.34	6.66 ± 0.56
MPNN (pos+vac)	7.07 ± 1.67	5.45 ± 0.38	6.16 ± 0.58
MPNN+LSTM (pos+vac)	7.06 ± 1.56	6.27 ± 0.81	6.50 ± 0.66
Multi-scale MPNN (IRIS)	5.53 ± 0.54	5.20 ± 0.22	6.60 ± 0.62
Multi-scale MPNN (IRIS, pos+vac)	**5**.**36** ± **0**.**51**	**5**.**18** ± **0**.**33**	**5**.**66** ± 0.50

Bold indicates best model. (vac) stands for using vaccination data and (pos) for using positive COVID cases.

**Figure 5:**
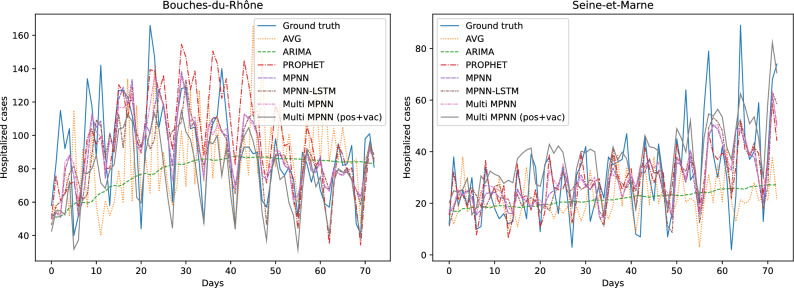
Number of patients in hospital due to COVID-19 per day for two departments of metropolitan France and predicted number of of patients in hospital by the different approaches.

Table 3 illustrates the average errors in terms of the number of hospitalized patients of the different approaches (along with standard deviations across the different tasks). We observe that the proposed models outperform the baselines on all three considered time resolutions (i. e., $3-$, $7-$ and $14-$days prediction). This demonstrates the usefulness of the considered features which the proposed models are build on (e. g., mobility data, vaccination rates, etc.). Surprisingly, we can see that very simple baseline such as AVG and AVG WINDOW are very competitive with sophisticated machine learning models. For instance, those two baselines outperform ARIMA and LSTM on all three considered time resolutions, while they also outperform some variants of the proposed model (especially in the case of long-term predictions). This highlights the complexity of the considered predictive task. Among the different baselines, standard MPNN and its variations are the best-performing ones, however, they are outperformed by at least one instance of the proposed model on all cases. Interestingly, we observe that providing the models with the number of fully vaccinated individuals results into slightly less accurate short-term and long-term predictions, but into more accurate mid-term predictions. More specifically, in the case of the 7 days ahead forecasts, the aforementioned feature offers MPNN and MPNN+LSTM absolute improvements of $0.56\%$ and $0.55\%$ in test error, respectively. A further improvement is achieved when the number of cases of the previous days is also given as input to the MPNN model for mid-term forecasts. The multi-scale variant that takes as input both department level and IRIS level graphs outperforms all the other approaches for short- and mid-term forecasts. When the model also utilizes the two aforementioned features, it quite surprisingly achieves lower errors for all short-, mid- and long-term forecasts. Besides the results of Table 3, we also chose again the same two departments (Bouches-du-Rhône and Seine-et-Marne) and visualized the predicted number of hospitalized patients (one day ahead prediction) by the different approaches. The results are illustrated in Fig. 5. Once again, we can see that AVG and ARIMA are the worst-performing methods. On the other hand, Prophet, MPNN, MPNN-LSTM and Multi-scale MPNN yield good predictive performance since they all predict the number of patients in hospital quite accurately. By manually inspecting Fig. 5, it is hard to determine which one of the above 4 models outperform the others in this task. This highlights the need for quantitative results such as those presented in Table 3.

**Figure 6:**
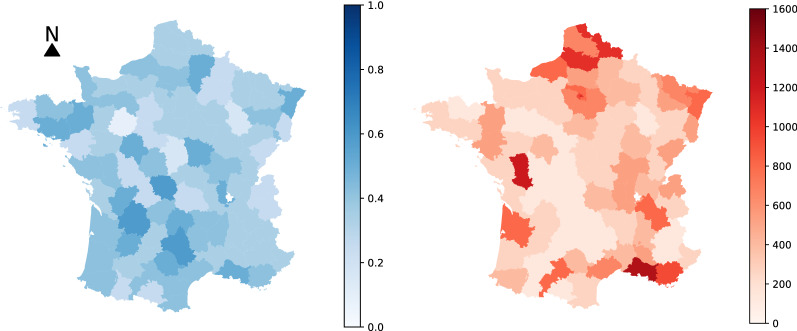
Maps of the departments of metropolitan France where the intensity of the color of each department is determined based on the relative test error on positive cases incurred by Multi-Scale MPNN (left) or the ground truth number of positive cases (right). The more blue a department, the larger the relative test error (as defined in Eq. (2)) on positive cases incurred by Multi-Scale MPNN (left). The more red a department, the higher the ground truth number of positive cases (right).

Figure 6 provides a qualitative evaluation of the proposed model. For each department, it illustrates the average number of cases per day (right) and also the relative error of the predicted number of cases. The relative error is defined as follows:

2 $$\begin{aligned} \text {relative error} = \frac{1}{n \tau }\sum _{t=T+1}^{T+\tau }\sum _{v\in V} \frac{|\hat{y}^{(t)}_v - y^{(t)}_v|}{y^{(t)}_v} \end{aligned}$$

where $y^{(t)}_v$ denotes the number of cases (or hospitalized individuals) in the department represented by node *v* on day *t*, and $\hat{y}^{(t)}_v$ denotes the predicted number of cases (or hospitalized individuals) for the same department and day. We observe that for departments with large numbers of cases (e. g., Île-de-France), the relative error is relatively small. On the other hand, high values of relative error occur mainly in departments whose average number of cases is low. This demonstrates that the proposed model can make more accurate predictions for departments heavily hit by the pandemic. This is particularly important since policymakers and public health officials usually put more focus on such regions, i. e., the ones hardest hit by the pandemic. On the other hand, regions where the outbreak is not that intense receive much less attention. For such regions, a large relative error might not be a serious problem. To make this clear, consider a department where in a given day, the actual number of cases is 10 and suppose that the predicted number of cases is 5. Even though the relative error is high, this has since the number of cases is relatively small. In the case of regions with large numbers of cases, a large relative error could be catastrophic since it would greatly enhance the risk of wrong decisions. As already discussed, for such departments, the relative error of the proposed approach is small. Hence, the predictions made by the model could enable governments and policymakers to make more informed decisions in order to halt the spread of the disease.

**Figure 7:**
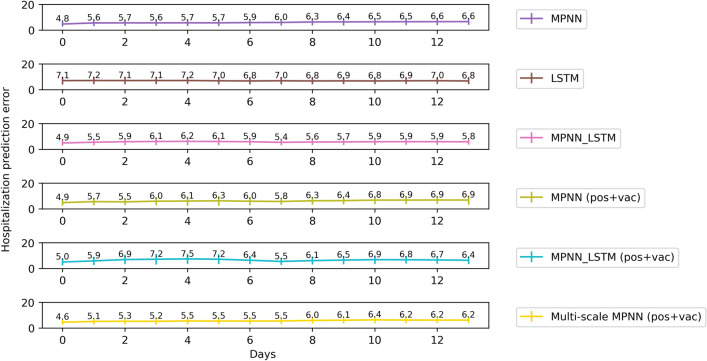
Average error (as defined in Eq. (1)) of the different methods for the number of hospitalized patients per day in the task of 14 days ahead forecast.

Figure 7 illustrates the average error of the proposed models and some of the baselines for each one of the next 14 days regarding hospitalization (long-term prediction task). It is clear that most instances of the proposed model achieve low levels of error in the first days, while the error gradually increases as we proceed further in the future. For instance, the multi-scale variant of the proposed model yields average errors of 4.6, 5.1 and 5.3 in the first three days, and errors of 6.2, 6.2 and 6.2 in the last three days (i. e., days 12 to 14). This is not surprising since the uncertainty of the model increases as time goes by because of the lack of observed data for the previous days. On the other hand, LSTM model variants achieve high errors for the whole forecasting period. Only the standard MPNN-LSTM approach manages to outperform our proposed model in long-term predictions, as opposed to it’s lower performance in short-term.

## Discussion

Understanding and controlling the mechanisms that govern spreading processes in complex networks is a fundamental task in many fields including disease spreading among others. The story of COVID-19 has highlighted the limitations of mathematical models. These models might fail to capture the full complexity of the contagion dynamics underlying infectious diseases. During such emergency situations, it is necessary to leverage all available tools that can provide a better understanding of the situation. Machine learning has recently emerged as a promising tool and has shown great potential in modeling such processes. Importantly, machine learning approaches can learn useful patterns directly from empirical data. Thus, they do not assume the contagion dynamics to be known in advance. Due to their flexibility, these methods have been recently employed to model the spread of the COVID-19 epidemic in different countries. Accurate machine learning models could help public health officials and policymakers to plan allocation of health care resources and to control the spread of the virus more effectively. In this work, we presented an instance of such a machine learning model that leverages population mobility data and other features to improve its predictive power. Message passing instances of GNNs can naturally model the interactions between regions, and can then combine this information with the one extracted from the input features. Then, the generated representations can be used for predicting the number of new cases as well as hospitalization rates. Even though the proposed models outperformed the baselines in our experiments, we observed that very simple baseline methods such as a method that predicts the average number of cases that occurred in the past *d* days achieve almost similar levels of performance with the proposed approach. This demonstrates that modeling contagion dynamics and especially predicting target variables related to them is a complex task even for very sophisticated learning algorithms. On the positive side, we observed that the proposed models yielded fairly accurate predictions for departments that have been severely hit by COVID-19. This is of critical importance for governments and other actors since they can capitalize on those predictions in order to adapt their policies and protocols, and plan ahead for the necessary preventive steps. We should note that other studies that also employ message passing models and leverage mobility data have reached similar conclusions, i. e., that for deep learning models, introducing additional mobility data improves results ^26,29,31^. Furthermore, with regards to the different models that are evaluated in this study, our results indicate that multi-scale models (i. e., Multi-scale MPNN) outperform the rest of the methods both in the task of predicting the number of cases and in the task of predicting the number of hospitalized patients. In the latter task, our results also suggest that vaccination data (given to the model as features) leads to further performance improvements. This has been confirmed by a very recent study which also showed that capturing the multi-scale and multi-resolution structures of graphs of regions is important to extract information that play a critical role in understanding the dynamics of COVID-19 ^30^.

Perhaps even more importantly than their predictive power, these models can provide explanations and identify which features have a strong relationship with the output variable. This can provide government officials and policymakers insights about the disease, e. g., the underlying factors contributing to the rapid spread of COVID-19. For instance, our analysis showed that population mobility has some impact on the spread of COVID-19. This is consistent with the findings of previous studies which showed that the probability that people living in a region are infected by the virus increases given more people moving in and out from that region ^35,39–41^. Other features led to similar conclusions. For instance, adding vaccination rates to the list of considered features generally improved the performance of the proposed models. This, for example, indicates that vaccination rate is indeed related to the spread of COVID-19. Furthermore, we found that more fine-grained approaches could also be more effective. This generally verifies our intuition that a person-to-person level analysis could lead to more accurate models and to better empirical results. However, since such information is not available, we instead consider the interactions between populations of individuals at different levels.

In terms of future directions of research, we need to mention that the proposed model can be easily extended to accommodate additional information. For instance, information about lockdown measures and weather conditions could be incorporated as node attributes. The spread of COVID-19 is known to critically depend on such features. Hence, we expect such features to improve the predictive performance of the proposed model. It would be also interesting to investigate whether those features are good indicators of the target variables (i. e., number of cases and hospitalization rates).

## Methods

### Models

Graph neural networks (GNNs) have recently become the standard approach for dealing with machine learning problems on graphs. These architectures can naturally model various systems of relations and interactions, including social networks ^42^ and particle physics ^43^. Importantly, GNNs have been employed in different application ranging from estimating the time of arrival in Google Maps ^44^ to discovering latent node information ^45^. The increasing activity in the field of GNNs has resulted into dozens of architectures being proposed in the past few years. Most of these architectures belong to the family of message passing neural networks ^46^. These models employ a message passing (or neighborhood aggregation) procedure to aggregate local information of vertices. Specifically, to update its representation, each node combines the representations of its neighbors with its own representation. Suppose we have a GNN model that contains *K* neighborhood aggregation layers. At each iteration ($k > 0$), the hidden state $\textbf{h}_v^{(k)}$ of a node *v* is updated as follows:

$$\begin{aligned} \begin{aligned} \textbf{a}_v^{(k)}&= \text {AGGREGATE}^{(k)} \Big ( \big \{ \textbf{h}_u^{(k-1)} :u \in \mathcal {N}(v) \big \} \Big ) \\ \textbf{h}_v^{(k)}&= \text {COMBINE}^{(k)} \Big (\textbf{h}_v^{(k-1)}, \, \textbf{a}_v^{(k)} \Big ) \end{aligned} \end{aligned}$$

where $\mathcal {N}(v)$ denotes the set of neighbors of node *v*, and $\text {AGGREGATE}$ is a permutation invariant function that maps the feature vectors of the neighbors of a node *v* to an aggregated vector. This aggregated vector is passed along with the previous representation of *v* (i. e., $\textbf{h}_v^{(t-1)}$) to the $\text {COMBINE}$ function which combines those two vectors and produces the new representation of *v*. Different $\text {AGGREGATE}$ and $\text {COMBINE}$ functions give rise to a different GNN. After *K* neighborhood aggregation steps, the representation $\textbf{h}_v^{(K)}$ of node *v* takes into account the entire *k*-hop neighborhood of *v*.

In this work, we use an instance of the above framework to compute the new representations of the regions. As already discussed, we construct two graph representations of regions at different granularities, namely IRIS areas and departments. The edge weights of the two graphs capture the mobility patterns, i. e., how many people moved from one region to another. Thus, each day *t* is associated with a pair of graphs $G_\text {DEP}^{(t)}$ and $G_\text {IRIS}^{(t)}$. For a sequence of days $1, \ldots , T$, we have two sequences of graphs, i. e., $G_\text {DEP}^{(1)}, \ldots , G_\text {DEP}^{(T)}$ and $G_\text {IRIS}^{(1)}, \ldots , G_\text {IRIS}^{(T)}$. The representations of the nodes of all those graphs are updated using a GNN. More specifically, we use the following neighborhood aggregation scheme to update the representations of the nodes:

$$\begin{aligned} \textbf{h}_v^{(k)} = f \bigg ( \sum _{u \in \mathcal {N}(v) \cup \{ v \}} \frac{1}{|\mathcal {N}(v)|+1} \textbf{W}^{k} \, \textbf{h}_u^{(k-1)} \bigg ) \end{aligned}$$

where $\textbf{W}^{k} \in \mathbb {R}^{d_k \times d_{k-1}}$ is a matrix of trainable parameters and *f* is a non-linear activation function such as ReLU. Note that in this setting, $\mathcal {N}(v)$ denotes the incoming neighbors of node *v*. Furthermore, following previous works ^47^, we compute a weighted sum of the messages received by the neighbors. Specifically, the sum of all weights is equal to 1 and is equally distributed among the neighbors of the node and itself. It should also be mentioned that the above update scheme integrates the $\text {AGGREGATE}$ and $\text {COMBINE}$ steps into a single function. Finally, note that for simplicity of notation, we have omitted the time index. The above model is in fact applied to all the input graphs $G_\text {DEP}^{(1)}, \ldots , G_\text {DEP}^{(T)}$ separately. A different model is applied to all the IRIS level graphs $G_\text {IRIS}^{(1)}, \ldots , G_\text {IRIS}^{(T)}$. In both cases, the weight matrices are shared across all graphs. Thus, we have two sets of weight matrices, i. e., $\textbf{W}_\text {DEP}^1, \ldots , \textbf{W}_\text {DEP}^K$ for the department level and $\textbf{W}_\text {IRIS}^1, \ldots , \textbf{W}_\text {IRIS}^K$ for the IRIS level.

As discussed above, as the number of neighborhood aggregation layers increases, the final node representations capture more global information. However, in some applications, local information might be equally useful or even more useful. Thus, to also explicitly retain this information, we concatenate the node representations that emerge at the different neighborhood aggregation layers $\textbf{h}_v^{(0)}, \ldots , \textbf{h}_v^{(K)}$ to obtain $\textbf{h}_v = \text {CONCAT} \Big (\textbf{h}_v^{(0)}, \ldots , \textbf{h}_v^{(K)} \Big )$, and the new representations can be regarded as vectors that encode multi-scale structural information, including the initial features of the node.

**Figure 8:**
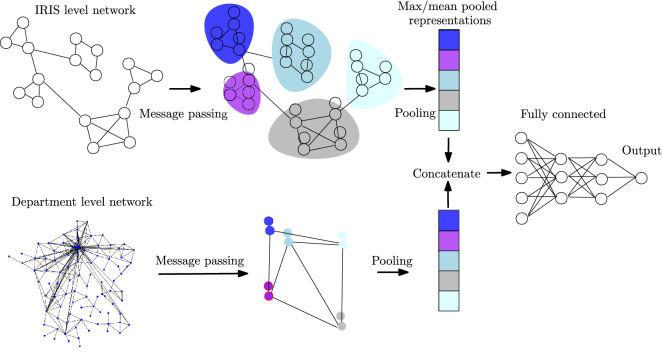
High-level illustration of our proposed multi-scale MPNN architecture. In the IRIS-level network each node is linked to an IRIS commune. In the department-level network each node represents a department. Each color is associated to a department.

Our objective is to generate a vector representation for each department and then feed this vector to a fully-connected network to compute the output. The GNN that is applied to the graphs whose nodes represent the different departments directly produce such representations $\textbf{h}_v^\text {DEP}$ (for the different days from 1 to *T*). The second GNN that is employed generates vector representations for the IRIS areas. Since a department consists of a set of IRIS areas, we generate a second representation for each department by aggregating the representations of the IRIS areas of which it is composed. Formally, let *v* be a node that represents a specific department in graphs $G_\text {DEP}^{(1)}, \ldots , G_\text {DEP}^{(T)}$, and let $\mathcal {S}(v)$ denote a set that contains all the IRIS areas associated with that department. Then, a second representation of node *v* is produced as follows:

$$\begin{aligned} \textbf{h}_v^\text {IRIS} = \frac{1}{|\mathcal {S}(v)|} \sum _{u \in \mathcal {S}(v)} \textbf{h}_u \end{aligned}$$

We thus compute the mean of the vector representations of the IRIS areas. We also experimented with a max aggregator. Then, the final representation of the department is computed as $\textbf{h}_v = \text {CONCAT} \Big (\textbf{h}_v^\text {DEP}, \textbf{h}_v^\text {IRIS} \Big )$. This representation is then passed onto one or more fully-connected layers to produce the output. For instance, if a single fully-connected layer is employed, the output is computed as follows:

$$\begin{aligned} \hat{y}_v = \text {ReLU} \big ( \textbf{W}_o \textbf{h}_v + \textbf{b}_o \big ) \end{aligned}$$

where $\textbf{W}_o \in \mathbb {R}^{1 \times d}$ and $\textbf{b}_o \in \mathbb {R}$ is a matrix of trainable parameters and the bias, respectively, while $\hat{y}_v$ is the number of predicted cases for the department represented by node *v* in the graph. We apply the $\text {ReLU}$ function to the output of the architecture since the number of new cases cannot take negative values. To train the model, we use the mean squared error as our loss function:

3 $$\begin{aligned} \mathcal {L} = \frac{1}{nT}\sum _{t=1}^T\sum _{v \in V} \Big (y_v^{(t)}-\hat{y}_v^{(t)} \Big )^2 \end{aligned}$$

where $y_v^{(t)}$ denotes the reported number of cases for region *v* at day *t*, *T* denotes the considered days, and *n* is equal to the number of departments (i. e., number of nodes of the department level graphs). An overview of the proposed architecture is illustrated in Fig. 8. Note that the proposed model is developed to extract patterns from spatiotemporal data. Spatiotemporal graphs consist of time-varying graph structure and features. In our setting, the graph structure depends on population mobility, while the features encode information such as the number of cases in the previous days, the vaccination rates, etc. The proposed MPNN model can deal with such spatiotemporal graphs since the different message passing layers are associated with discrete points in time and can capture the temporal aspects of the input data.

To update the representations of the nodes of the department level graphs, besides the model described above (MPNN), we also follow a commonly-used approach in time series forecasting (MPNN-LSTM). Given a sequence of graphs $G_{\text {DEP}}^{(1)}, \ldots , G_{\text {DEP}}^{(T)}$ that correspond to a sequence of dates, we utilize a message passing model at each time step, to obtain a sequence of representations $\textbf{h}_v^{(1)},\ldots ,\textbf{h}_v^{(T)}$ for each node *v* representing some department. These representations are then fed into a Long-Short Term Memory network (LSTM) ^48^ which can capture the long-range temporal dependencies in time series. We expect the hidden states of an LSTM to capture the temporal information and nonlinear dynamics from the historical data of COVID-19. The new representation of a given region is the hidden state of the last time step of the LSTM model. These representations are then passed on to fully-connected layers to produce the output.

### Experimental setup

We next present the values of the hyperparameters of the proposed models. We set the number of epochs to 500 epochs with early stopping applied when the validation accuracy does not increase for 50 epochs. Early stopping starts to occur from epoch 100 and onward. The batch size is set to 8. We used the Adam optimizer with a learning rate of $10^{-3}$. We set the number of hidden units of the message passing layers to 64. Each message passing layer is followed by a batch normalization layer and a dropout layer with a dropout ratio of 0.5. The model that achieved the highest validation accuracy is stored in the disk and is then retrieved and used to make predictions for the test samples. For the MPNN+LSTM model, the dimension size of the hidden states of the LSTM is set equal to 64.

### Baselines

We compare the proposed models against the following 6 baselines and benchmark methods, which have been applied to the problem of COVID-19 forecasting: (1) AVG: The average number of cases for the specific region up to the time of the test day; (2) AVG WINDOW: The average number of cases in the past *d* for the specific region where *d* is the size of the window; (3) LAST DAY: The number of cases in the previous days is the prediction for the next days; (4) LSTM^49^: A two-layer LSTM that takes as input the sequence of new cases in a region for the previous week; (5) ARIMA^50^: A simple autoregressive moving average model where the input is the whole time-series of the region up to before the testing day; and (6) PROPHET^51^: A forecasting model for various types of time series whose input is similar to that of ARIMA.

## Supplementary Information

Supplementary Information 1.

Supplementary Information 2.

Supplementary Information 3.

## Data Availability

Hospitalization, positivity, vaccination, and IRIS datasets are provided as supplementary material. They are also available publicly at data.gouv.fr and from the corresponding author on reasonable request. The mobility data that support the findings of this study are available from dataforgood.facebook.com but restrictions apply to the availability of these data, which were used under license for the current study, and so are not publicly available.
